# The Structure of Human Neuromuscular Junctions: Some Unanswered Molecular Questions

**DOI:** 10.3390/ijms18102183

**Published:** 2017-10-19

**Authors:** Clarke R. Slater

**Affiliations:** Institute of Neuroscience, Newcastle University, Newcastle upon Tyne NE1 7RU, UK; c.r.slater@ncl.ac.uk; Tel.: +44-191-222-5954

**Keywords:** neuromuscular junction, structure, neuromuscular transmission, human, mouse, disease

## Abstract

The commands that control animal movement are transmitted from motor neurons to their target muscle cells at the neuromuscular junctions (NMJs). The NMJs contain many protein species whose role in transmission depends not only on their inherent properties, but also on how they are distributed within the complex structure of the motor nerve terminal and the postsynaptic muscle membrane. These molecules mediate evoked chemical transmitter release from the nerve and the action of that transmitter on the muscle. Human NMJs are among the smallest known and release the smallest number of transmitter “quanta”. By contrast, they have the most deeply infolded postsynaptic membranes, which help to amplify transmitter action. The same structural features that distinguish human NMJs make them particularly susceptible to pathological processes. While much has been learned about the molecules which mediate transmitter release and action, little is known about the molecular processes that control the growth of the cellular and subcellular components of the NMJ so as to give rise to its mature form. A major challenge for molecular biologists is to understand the molecular basis for the development and maintenance of functionally important aspects of NMJ structure, and thereby to point to new directions for treatment of diseases in which neuromuscular transmission is impaired.

## 1. Why Studying the Structure of Human Neuromuscular Junctions (NMJs) Is Important

The ability to move purposefully within the environment is an essential feature of the life of animals, including humans. The neuromuscular junction (NMJ) is the site where the central nervous system CNS interacts with skeletal muscle fibers and causes them to contract—where intent and purpose are converted into action. During the process of neuromuscular transmission, patterned electrical activity in a motor neuron causes the release from its terminals of a chemical transmitter which then acts on the nearby surface of the target muscle cells to cause localized electrical depolarization and the activation of contraction. Anything that impairs this complex process of signal transmission is likely to lead to muscle weakness or paralysis, and potentially to a profound deterioration of the quality of life.

Many pathological conditions which are characterized by impaired neuromuscular transmission are known in humans, including those caused by mutations in more than 30 genes [1]. For many of these conditions, the available treatments are far from ideal. Efforts to improve treatment of patients with these conditions depend on understanding the factors that normally determine the efficacy of NMJ function. These factors include not only the functioning of the individual protein molecules and molecular complexes that mediate transmitter release and action, but how those complexes are localized within the complex structure of the NMJ. As a result, mutations may exert their pathogenic effect not only by altering the abundance and function of the proteins encoded by the mutated genes, but also by altering various aspects of NMJ structure. While much has been learned in recent years about the molecular basis of transmitter release and action, we still remain largely ignorant of how the structural features of the NMJ are determined.

In this brief review, my aim is to interest molecular scientists in some of the fundamental questions associated with trying to understand how the structure of the NMJ is regulated, how it evolved, and how it responds to pathological processes. I start by reviewing the basic properties of the NMJ and neuromuscular transmission. I then consider briefly how human NMJs fit into the broad spectrum of NMJs in the animal kingdom, identifying some distinctive feature of human NMJs, and consider how those features may influence the response of human NMJs to disease processes. In the final section, I consider what is currently known, but mostly not known, about the molecular basis of these functionally significant structural features. The references cited in this review are a representative sample, rather than a comprehensive catalog of all published work of relevance.

## 2. Basic Properties of the NMJ

### 2.1. NMJ Structure

**Figure 1:**
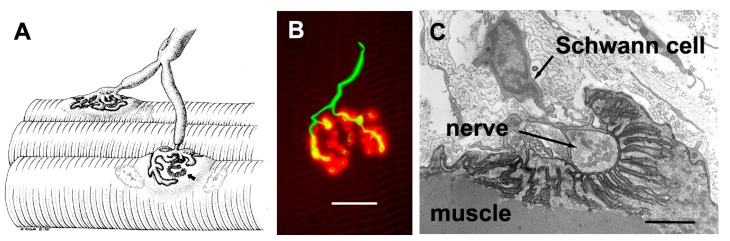
Mammalian neuromuscular junctions (NMJ) structure. (**A**) Diagram showing a motor axon which branches to innervate two muscle fibers at the NMJs. Note the extensive branching of the motor axon terminal. Arrow shows region where nerve has been removed, revealing postsynaptic folds (From Salpeter, 1986, with permission) [2]; (**B**) Human NMJ showing the nerve terminal (green, immunolabeling of synaptophysin and neurofilament protein) and the postsynaptic acetylcholine receptors (AChRs) (red, fluorescent tagged α-bungarotoxin). Note the swollen “boutons” of the nerve terminal, from which transmitter is released. Scale bar, 20 μm; (**C**) Electron micrograph of a section through a single bouton. Note the extensive infolding of the postsynaptic muscle fiber membrane. Scale bar, 1 μm.

The axons that control skeletal muscle cells arise from the centrally located cell bodies of the motor neurons and travel, generally unbranched, to the target muscles. There they branch profusely, and make contact with many muscle fibers. The sites of nerve–muscle contact are highly specialized (Figure 1). The fine (0.1 μm) motor nerve terminal forms numerous varicosities (“boutons”), usually 1–5 μm across, from which transmitter is released (Figure 1B). As revealed by electron microscopy, in addition to many mitochondria and arrays of endoplasmic reticulum, the nerve terminal contains many 50 nm diameter, membrane-bound synaptic vesicles (SVs) which contain the transmitter. The SVs are normally particularly concentered around electron-dense structures in the presynaptic membrane, known as the “active zones”, which are the sites of evoke transmitter release (Figure 1C). The boutons adhere to the muscle cell surface, leaving a gap of about 50–100 nm which contains extracellular matrix, often taking the form of a discrete basal lamina (BL). They are capped by several non-neural Schwann cells.

The postsynaptic surface of the muscle cell harbors a very high density of transmitter receptors. These are transmembrane, ligand-gated ion channels which are usually associated with arrays of accompanying membrane and cytoskeletal proteins [3]. In vertebrates, where the transmitter is acetylcholine (ACh), the ACh receptors (AChRs) form a near-crystalline array in the muscle fiber membrane where the density is about 10,000 AChRs/μm^2^ [4]. In the EM these appear as membrane regions of high electron-density. In vertebrates, the postsynaptic membrane is folded, with the folds extending up to 1 μm or so into the cytoplasm. These folds increase the area of postsynaptic membrane by a factor of 2–8, depending on the species (Figure 1C).

### 2.2. NMJ Function

The essential role of the NMJ is to convert a temporal sequence of action potentials (APs) in motor neurons into muscle contractions. The molecular events that cause muscle contraction are triggered by increases in the intracellular calcium concentration. This, in turn, is triggered by depolarization of the plasma membrane beyond a critical threshold of about −50 mV, which opens voltage-gated calcium channels. It is the job of the motor neuron to bring about that initial depolarization.

**Figure 2:**
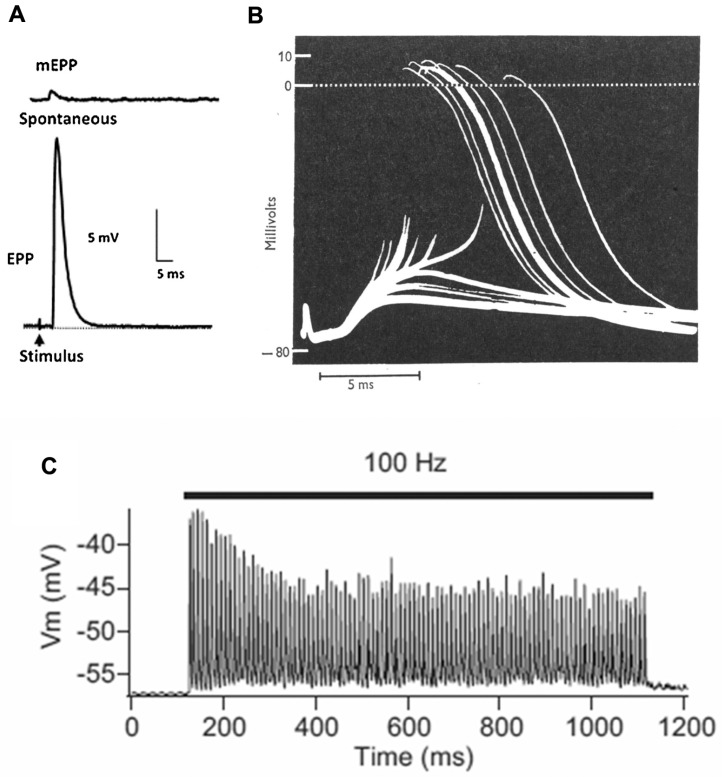
Basics of neuromuscular transmission. (**A**) Intracellular recordings of synaptic events at a mouse NMJ: **top**, miniature endplate potential (mEPP), the response to a single quantum of ACh; **bottom**, plate potential (EPP), the nerve-evoked response to about 50 quanta; (**B**) Intracellular recordings from an NMJ from a human patient with myasthenia gravis. The smaller events are subthreshold EPPs, which result from the low numbers of AChR in this disease. (From Elmqvist et al., 1964, with permission) [5]. The large events, of nearly constant amplitude, are mAPs evoked by those EPPs that reach threshold; (**C**) A train of EPPs at a mouse EPP in which the muscle cell action potential (mAP) is blocked with μ-conotoxin. During stimulation at 100 Hz, there is a decline in EPP amplitude. (Adapted from Ruiz et al., 2011, with permission) [6]. However, in the absence of the toxin, the EPPs would all be large enough to trigger mAPs.

The basic process of neuromuscular transmission is similar in most animals (Figure 2). The transmitter, which in mammals is acetylcholine (ACh), is stored in the SVs in packets of about 10,000 molecules (in vertebrates). These packets are often referred to as “quanta”. Exocytosis of a single SV, which happens spontaneously at a rate of about 1 Hz, allows the transmitter to diffuse within the 50–100 nm wide cleft between nerve and muscle. It then binds to ligand-gated cation channels, the ACh receptors (AChRs). These channels then open, typically for about 1 ms, allowing a transient net influx of (usually) positive ions into the muscle cell, thereby reducing the normally negative “resting potential” (−75 mV) by about 1 mV (Figure 2A).

Depolarization of the nerve terminal by an action potential (nAP) opens voltage-gated calcium channels, resulting in an increase in the intracellular free calcium level. This triggers the near simultaneous release of a number of transmitter quanta whose effects sum, giving rise to a brief depolarization of about 35 mV, the endplate potential (EPP). This transient “depolarization” either triggers contraction directly (as in many invertebrates), or (as in vertebrates) triggers a new AP in the muscle cell (mAP), which propagates along the muscle fiber triggering contraction as it goes (Figure 2B). In mammals, the number of quanta released by an individual nAP (the “quantal content”—QC—of the response) during low frequency activity (0.1–1 Hz) is normally 2–4 times greater than required to trigger a mAP, the basis of a high “safety factor” of transmission [7]. However, during higher frequency activity (10–100 Hz), as in vivo, the QC and EPP amplitude decline, though normally remaining adequate to activate the target muscle cell (Figure 2C). The recording in Figure 2B is from a patient with myasthenia gravis, in which the EPP is abnormally small as a result of autoantibody attack on the AChRs, and frequently fails to trigger a mAP, thus revealing the underlying EPP.

### 2.3. Functional Organization of the Vertebrate NMJ

#### 2.3.1. Presynaptic

The efficacy of neuromuscular transmission depends critically on the number of transmitter quanta released from the nerve by each nAP [8]. Many factors influence that number. One of these is the spatial extent of specialized nerve–muscle contact. In general, the greater the area of contact, the more quanta are released, with a ratio in vertebrates of about 0.15 quanta/μm^2^ during stimulation at a frequency of about 1 Hz. Large diameter muscle fibers have lower electrical resistance, and therefore require more ACh-induced current to generate an EPP which reaches the mAP threshold. Consistent with this, NMJs on large muscle fibers are generally larger than those on small fibers. However, as detailed below, human NMJs represent a surprising exception to this “rule”.

**Figure 3:**
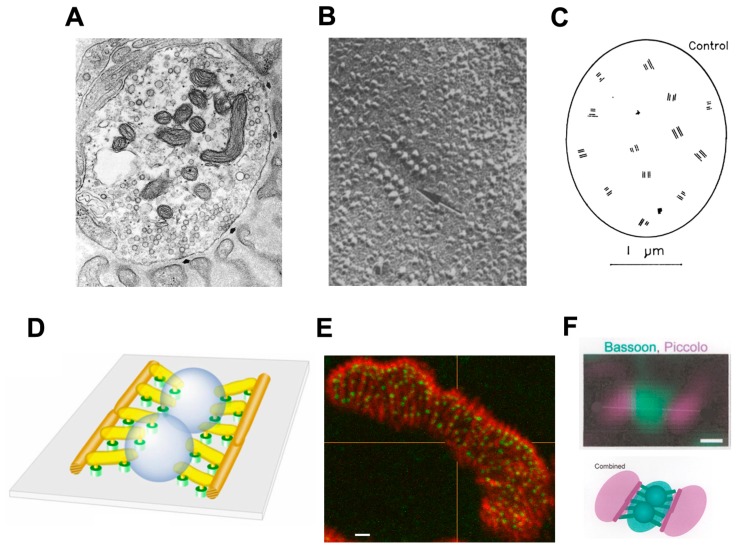
Vertebrate Active Zones (AZs). (**A**) EM image of terminal bouton, with prominent mitochondria and SVs. The arrowheads point to two AZs. Note the clusters of synaptic vesicles (SVs) around the AZs (indicated by arrows). (From Engel, 2004, with permission) [15]; (**B**) A mammalian AZ, indicated by arrow, visualized in a freeze-fracture replica. Note that characteristic double rows of intramembranous particles, believed to include the Ca_v_2.2 channels. Scale bar, 0.5 μm. (From Fukunaga et al., 1983, with permission) [16]; (**C**) Diagram of freeze-fracture replica of a nerve terminal bouton showing the distribution of AZs. Scale bar, 100 nm. (From Fukunaga et al., 1982, with permission) [13]; (**D**) Fine structure of mouse AZ determined by 3D electron microscope tomography. Note the numerous, regularly arranged structural components and the two SVs held in a central position. (From Nagwaney et al., 2009, with permission) [9]; (**E**) Fluorescence image of part of a mouse motor nerve terminal. AChRs (red, α-bungarotoxin), bassoon, a protein component of the AZ (anti-bassoon, green). Scale bar, 100 nm. (From Chen, 2012, with permission) [17]; (**F**) Images of bassoon (green) and piccolo (red) distribution at a single mouse AZ, recorded by STED microscopy. Below, representation of the STED labeling superimposed on a simplified rendition of the AZ structure as in (**D**). Scale bar, 50 nm. (From Nishimune et al., 2016) [11].

The release of transmitter quanta occurs at “active zones” (AZs), highly ordered multimolecular complexes in the presynaptic membrane (Figure 3). 3D electron microscope tomography has revealed a large number of distinct structural components of vertebrate AZs [9,10]. Immunolabeling studies, in parallel, have identified a number of the molecular components of the AZ and recent studies using super-resolution microscopy have begun to reveal where those molecules are located within the structure of the AZ [11]. A particularly important component of the AZs are the voltage-gated calcium channels (Ca_V_s) that mediate quantal release. *En face* views of the presynaptic membrane, as obtained in freeze-fracture electron microscopy or by high resolution immunofluorescence microscopy after suitable labeling, show that the AZs are distributed very non-randomly, with a mean inter-AZ spacing of about 0.5 μm but few closer to their neighbor than about 0.3 μm (Figure 3) [12,13,14]. The spacing of AZs is very similar in all species that have been studied in detail. This may well be to reflect the need to ensure that increase in Ca^2+^ concentration resulting from the Ca^2+^ ions entering at one AZ have relatively little effect on events at adjacent AZs.

#### 2.3.2. Postsynaptic

**Figure 4:**
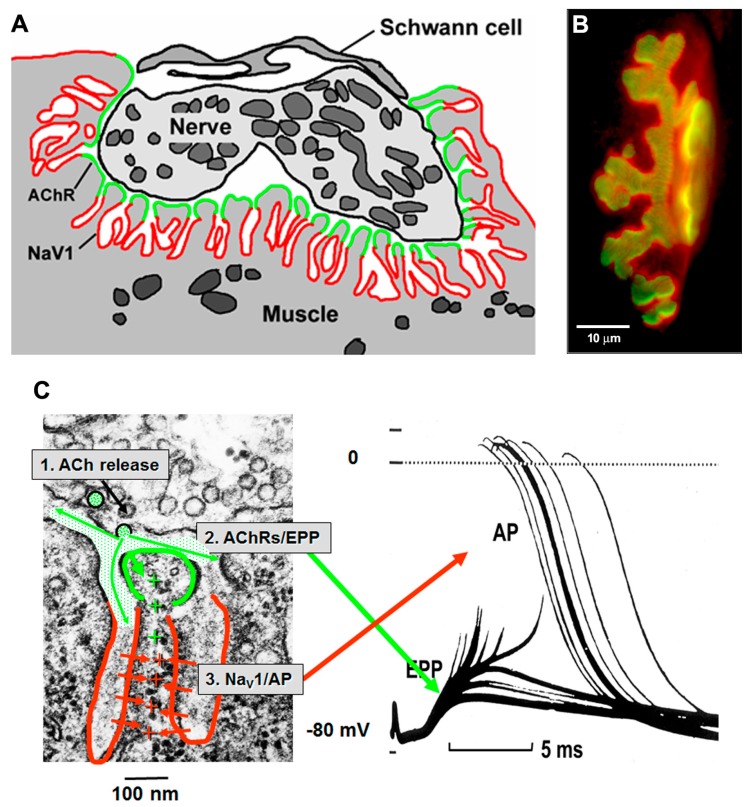
Postsynaptic folds and the anatomy of mAP generation. (**A**) Diagram of ion channel distribution at a rat NMJ. AChRs (green) are concentrated at the crests of the folds, while Na_V_1.4 channels are concentrated in the depths of the folds; (**B**) Fluorescence of rat NMJ showing AChRs (green) and Na_V_1.4 channels (red). Note that the Na_V_1.4 labeling protrudes beyond the AChRs because the Na_V_1.4 channels are located in the depths of the folds; (**C**) Anatomy of mAP generation. (1) ACh release from the nerve occurs next to the region of high AChR density (green); (2) This allows positive ions to enter the muscle, giving rise to the EPP; (3) The depolarization of the EPP opens the Na_V_1.4 channels in the depths of the folds, allowing a much bigger influx of positive ions which triggers that mAP. (From Slater, 2017, with permission) [21].

The AChRs are concentrated at the crests of the postsynaptic folds, adjacent to the nerve [18]. The membrane in the depths of the folds contain a high density of the voltage-gated sodium channels (Na_V_1s) that mediate the initiation of the AP [19,20]. In vertebrates, the AZs are normally aligned with the openings of the underlying postsynaptic folds, so that transmitter quanta are released into the mouth of the folds (Figure 4). This gives optimal access to the AChRs clustered at the crests, and part way down the walls, of the folds. During transmission the current resulting from ACh action, which enters the folds at their crests, flows into the core of the muscle fiber through the narrow, high resistance, sheets of cytoplasm that lie between the folds. This current is optimally located to depolarize the membrane of the folds and thereby to open the Na_V_1s located within them, thus initiating the mAP. As a result, the folds act as amplifiers of transmitter action.

#### 2.3.3. Synaptic Cleft

Termination of ACh action occurs when ACh unbinds from the AChRs. It is then rapidly hydrolyzed by the action of acetylcholinesterase (AChE) which is bound to the BL in the synaptic cleft. The choline thus released is taken back into the nerve terminal by a specific uptake protein and reused to synthesize new transmitter within the nerve terminal.

## 3. An Evolutionary Overview of the Human NMJ

Although NMJs in most species throughout the animal kingdom share many important features, there are also important differences [8,22]. Human NMJs, in particular, appear to occupy one end of a range of NMJ “plans”. To appreciate the significance of these differences, it helps to consider some of the most important ways in which NMJs have changed during evolution.

### 3.1. Patterns of Innervation

Most animals can be placed in one of three groups, depending on their basic body plan. Worm-like animals (e.g., earthworms, planarians), molluscs and nematodes maintain their form by virtue of a hydrostatic skeleton. They usually have many small, mononucleated muscle cells which operate as functional syncytia. Their muscles are generally innervated by extended motor axons that terminate close to the muscle cells. An important exception to this rule are the Nematodes, like *Caenorhabditis elegans*, whose long, electrically excitable, muscle “arms” make contact with the electrically inexcitable motor axons which run in dorsal and ventral nerve cords [23].

Arthropods (e.g., crustaceans and insects) have a semi-rigid external skeleton with joints that have a single main axis of rotation which can be controlled by relatively few muscles. Their electrically inexcitable muscle cells are usually long, multinucleated fibers. These are innervated by a small number of motor axons which make bouton-like contacts at many points along the length of the muscle fiber. This “distributed innervation” ensures that the whole length of the muscle fiber can be activated by a single nAP.

Vertebrates (e.g., fish, amphibians, reptiles, birds, mammals) have an internal skeleton whose joints often have multiple axes of rotation, and are controlled by the action of many different muscles. As in arthropods, they have long, multinucleated muscle fibers. A crucial feature of vertebrate evolution was the emergence of an electrically excitable muscle fiber membrane, capable of generating propagating mAPs. As a result, the nerve can activate the whole fiber efficiently from a single site of contact. This ‘focal innervation’ makes very efficient use of the amount of ‘neuronal resource’ available.

### 3.2. Vertebrate Trends

**Figure 5:**
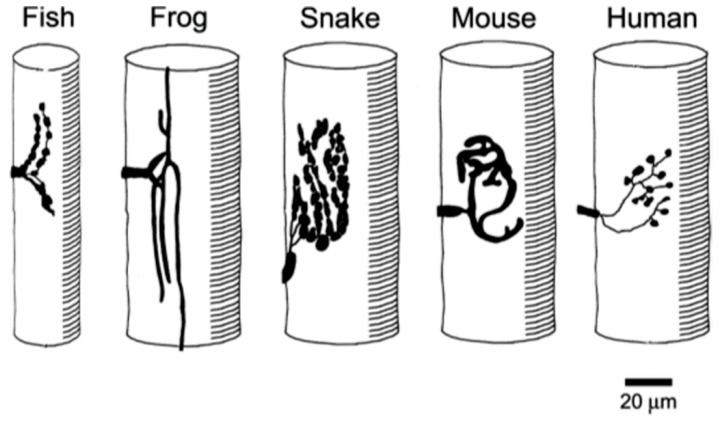
Patterns of vertebrate muscle innervation. Diagrams, based on nerve-specific stains, of the distribution of the motor nerve terminals in a range of vertebrate species. Note the variety of sizes and conformations, with human terminals among the smallest.

In addition to the broad, inter-Phylum, trends just described, there are also clear trends within the Vertebrates. The conformation of vertebrate NMJs varies considerably from species to species (Figure 5).

**Figure 6:**
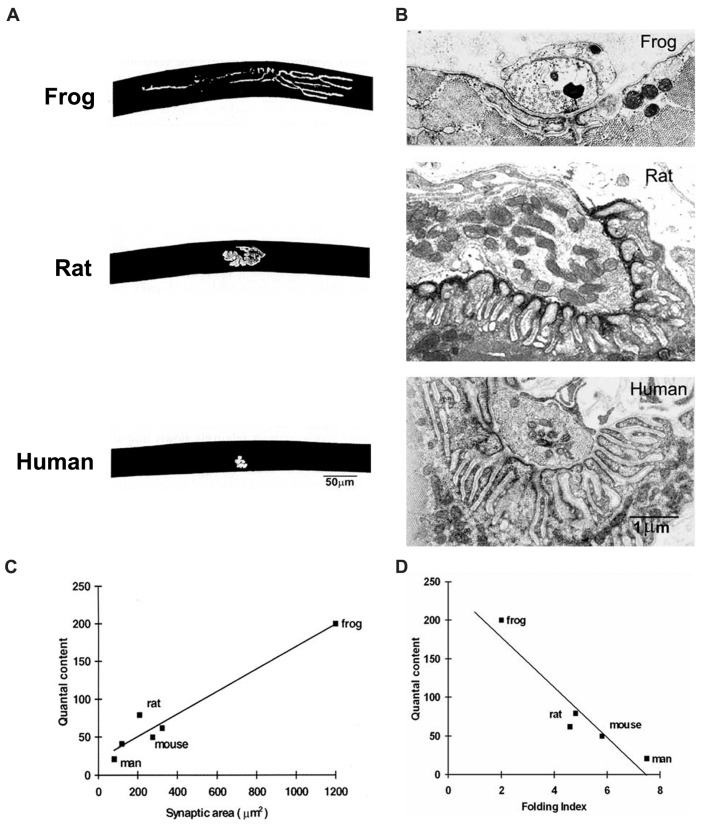
Correlations of structure and function in vertebrate NMJs. (**A**) Fluorescence images of frog, rat and human NMJs, labelled with α-bungarotoxin. Note that the human NMJ is much smaller than the other two; (**B**) EM images of NMJs in the same species. Note that the extent of postsynaptic folding increases from frog to human; (**C**) Quantal content is related to synaptic area in the vertebrate species studied; (**D**) Quantal content is related inversely to the extent of folding (folding index, FI).

As one progresses from fish to mammals, there is an increase in the focusing of the NMJ at a single site and an increase in the extent of postsynaptic folding (Figure 6). For those species that have been studied in detail, it is clear that while there is a clear correlation between the QC and the size of the NMJ, there is a roughly inverse correlation between the QC and the extent of folding. This is consistent with the view that the folds amplify transmitter action, so that more extensive folds compensate for smaller nerve terminals, thus maintaining the overall efficacy of transmission.

### 3.3. The Human NMJ

Human NMJs appear to occupy one end of the spectrum of vertebrate NMJs that have been studied (Figure 6). Even though human muscle fibers have quite large diameters (50 vs. 30 μm in mice), human NMJs are among the smallest vertebrate NMJs that have been described. Consistent with the relatively constant relationship of QC and synaptic area, the QC at human NMJs is only about 20–30, while that at mouse NMJs is typically 50–100.

At the same time, human NMJs also have very extensive folding, which increases the local area of membrane about 8-fold. In extreme cases, the folds are separated by sheets of cytoplasm less than 0.2 μm thick, which is occupied by periodic densities suggestive of molecular links between the folded membranes (cf. Figure 1). As mentioned above, this extreme folding may reflect a compensatory enhancement of postsynaptic amplification of ACh action. At present there is no clear understanding of why human NMJs should have acquired this particular set of features. One possibility is that in large animals, the number of muscle fibers innervated by each motor neuron tends to be greater than in small animals. It may thus be more economical of cellular resources to have small nerve terminals, and to compensate by enhancing postsynaptic amplification.

## 4. Human NMJs and the Response to Disease

Whatever the biological explanation for the distinctive properties of human NMJs may be, it is worth considering what impact those features are likely to have on the response of human NMJs to disease processes. Diseases that cause impairment of neuromuscular transmission have been known for many years [24]. However, even the best known and most common of these, myasthenia gravis, has a prevalence of only 15/100,000, probably reflecting the vital role played by the NMJ in survival. There are two main classes of NMJ disease; sporadic, mediated by autoantibodies which attack NMJ components, and inherited, mediated by mutation of genes encoding proteins which play functionally significant roles at the NMJ. Here only a brief overview of these conditions is appropriate, but excellent, more detailed, reviews can be found elsewhere [1,25,26].

The autoimmune forms target proteins which have significant extracellular domains, most notably the Ca_V_2 channels in the nerve terminal (Lambert-Eaton Myasthenic Syndrome) and the AChRs in the postsynaptic membrane (myasthenia gravis), along with proteins that influence its distribution, including agrin, LRP4 and MuSK. In addition to causing a reduction in abundance of these key proteins, the autoantibodies that bind to them may also cause local, complement-mediated, destruction of cellular structures such as the postsynaptic folds.

The inherited diseases of the NMJ, known collectively as congenital myasthenic syndromes (CMS), affect a much wider range of proteins. Recent studies have identified more than 30 genes known to be loci for mutations that are pathogenic for the NMJ [1]. The most common target is the AChR, and mutations of the genes encoding the four classes of AChR subunit lead to changes in channel kinetics, both speeding up and slowing down, as well as to decreases in AChR abundance. Mutations leading to the “slow channel syndrome” cause local cytotoxicity, including structural disruption of the postsynaptic membrane, mediated by Ca^2+^ entry during prolonged ACh action. A similar effect arises from mutations of the gene encoding ColQ, the collagenous tail which binds AChE molecules to the synaptic BL. Other mutations cause a decrease in the abundance of the AChRs. These often lead to alterations of the overall size and conformation of the NMJ (see below). There are many additional proteins associated with the AChRs which act together to maintain the high-density AChR cluster at the NMJ. These include rapsyn, agrin, MuSK, Lrp4 and DOK-7. Mutations of the genes encoding these proteins also typically cause a reduction in AChR clustering and an associated weakening of transmission. In recent year, a number of mutations in presynaptic proteins, which alter transmitter release from the nerve terminal, have also been identified. These proteins act either to synthesise or package ACh (CHAT, VAChT), or to mediate its release from the nerve (e.g., SYT2, VAMP1, SNAP25B, MUNC18-1).

Many of the conditions which cause impaired neuromuscular transmission are also associated with structural changes of the NMJ. In the following sections I will consider two of the most common of these, reduced NMJ size and QC (Presynaptic factors), and their very extensive folds (Postsynaptic factors).

### 4.1. Presynaptic Features

The low QC of human NMJs make them particularly susceptible to disease processes that impair release. Largely because the relatively small nerve terminals typically release only 20–30 quanta in response to each nAP, the safety factor of transmission is only about 2 during low frequency stimulation and likely lower still during intense activity [7]. By contrast, the safety factor at rodent NMJs is about 4, so they can continue to function even in the presence of a 50% reduction in transmitter output. These properties make the human NMJ particularly susceptible to any factors that reduce QC still further.

#### 4.1.1. Quantal Release

There are two main causes for a pathological reduction in QC; a reduction in QC per unit area of synaptic contact (QC/area), and a reduction in the area itself. One class of conditions in which QC/area is reduced are those in which the entry of Ca^2+^ into the nerve terminal, evoked by the nAP, is reduced. The best known example of this is the Lambert-Eaton Myasthenic syndrome in which attack by autoantibodies to the Ca_V_2.2 channels in the nerve terminal cause a reduction in the abundance of those channels [27]. A second class are those in which Ca^2+^ entry is normal, but mutations in key proteins o the exocytosis complex result in much reduced quantal release [28,29].

In other conditions, QC appear to be reduced because the NMJs are abnormally small, rather than because of any impairment of the molecular events in release. For example, mutations of the *DOK7* gene, which encodes an essential activator of the muscle specific kinase (MuSK), a key organizer of postsynaptic differentiation, are associated with a 50% reduction in the area of synaptic contact and quantal content at the NMJs and, hence, a very significant impairment of neuromuscular transmission [30,31]. A similar reduction is seen in some other examples of inherited NMJ disease [31,32,33]. These conditions raise important questions about how NMJ size is determined.

#### 4.1.2. Nerve Terminal Size

The factors which determine the extent of synaptic area at individual NMJs are poorly understood. An important feature of NMJs is their “homeostatic plasticity”, i.e., the ability to respond to a variety of situations in a way that helps to maintain a constant efficacy of neuromuscular transmission [34]. An important aspect of this plasticity is the tendency of the NMJs of humans and other mammals to respond to impairment of transmission by producing axonal sprouts which grow away from the original NMJ and form new sites of synaptic contact [35]. This outgrowth is usually both preceded, and enabled, by sprouting from the terminal Schwann cells, which thus play an important role in the “self-maintenance” of the NMJ [36].

**Figure 7:**
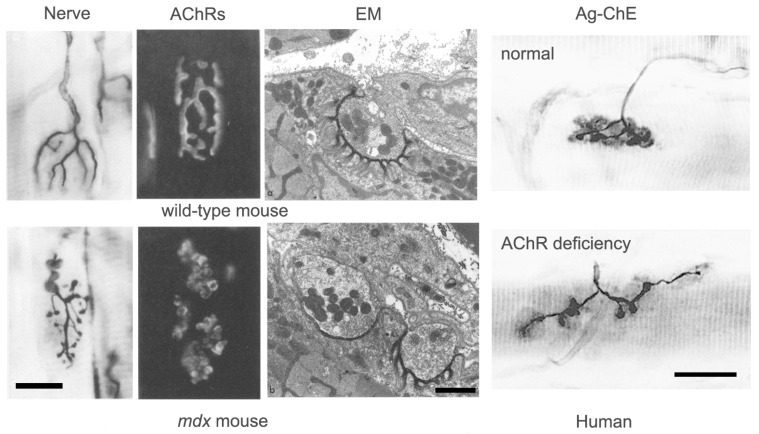
Pathological features of NMJs. The images of mouse NMJs show, from left to right, the nerve, the distribution of AChRs and the ultrastructure. The upper images show NMJs from wild-type mice while the lower images show NMJs from dystrophin-deficient *mdx* mice, in which most muscle fibers degenerate, and then regenerate. Note the dramatic change in the conformation of the nerve terminal and the associated AChRs in the *mdx* mice, reflecting a new pattern of innervation established when the nerve reinnervates regenerated muscle fibers. In the EM images, note the presence of region of normal folding, presumed to be original contacts, adjacent to a region devoid of folding, presumed to be a new contact. Scale bars, light-micrographs, 20 μm, EM images, 1 μm. (From Lyons and Slater, 1991, with permission) [39]. The images of human NMJs are from a normal patient (**top**) and a patient with inherited AChR deficiency (**bottom**). Scale bar, 20 μm. (From Slater et al., 1997, with permission) [37].

Good examples of this homeostatic response are seen in patients with congenital AChR deficiency (Figure 7) [37,38]. In these patients, a reduction in AChR density at the NMJ results in decreased mEPP and EPP amplitude, but no decrease in QC. The NMJ is clearly elongated and fragmented, however the area of synaptic contact is normal. In these patients, it appears that impaired transmission has induced axonal sprouting and new synapse formation, but that this has only partially compensated for the reduced AChR abundance. By contrast, in patients with mutations of the DOK7 gene, which encodes a key postsynaptic signaling molecule, although transmission is impaired, the NMJs are abnormally small [31]. Little is currently known about why homeostatic plasticity is effective in some conditions and not in others.

#### 4.1.3. NMJ Conformation

It is notable that the response of mammalian NMJs to disease often results in a striking change in their conformation. In the absence of disease, the NMJs of different vertebrate species have consistently different shapes and sizes (cf. Figure 5). These differences are strikingly exemplified by comparison of the elongate NMJs of frogs with the much more compact NMJs of mammals. Just as consistent, but more subtle differences exist between the NMJs of different muscles in the same animal. Thus, in mammals, many NMJs consist of curved bands of postsynaptic differentiation which form along the terminal branches of the motor axon, suggesting the appearance of a pretzel. Others, by contrast, consist of numerous discrete, spot-like contacts, which give the NMJ a more “fragmented” appearance. It is not obvious, a priori, that these difference conformations would have any impact on NMJ function.

As a result of the nerve sprouting often induced by disease, mammalian NMJs often tend to become increasingly “fragmented” and elongated. In some ways, these “adapted” NMJs resemble those in lower vertebrates which are associated with “distributed” innervation. The extent of “fragmentation” of NMJs has recently received much attention as a feature of disease and aging. However, although increased fragmentation has sometimes been taken as a sign of NMJ deterioration or disintegration, as with the differences in NMJ conformation between muscles in the same species, there is no compelling evidence that an increase in fragmentation per se has any functional consequence [40].

### 4.2. Postsynaptic Features

The extensive postsynaptic folds of human NMJs are also targets of a number of disease processes. These include both autoimmune conditions, in which autoantibodies are directed against molecular components of the NMJ, and genetically determined ones which are characterized by excessive opening of postsynaptic cation channels which allow cytotoxic increases in Ca^2+^ concentration within the postsynaptic cytoplasm [41]. In diseases of both sorts, the orderly array of folds is often disturbed resulting in a reduction in the regularity and depth of the folds and in the total amount of folded membrane. Given the presumed role of the folds as amplifiers of transmitter action, it is clear that such a disturbance, on its own, would be expected to raise the electrical threshold for generating a mAP and thereby reduce the overall safety factor for transmission. In humans, the apparently particularly important role of the folds in ensuring reliable transmission may make the functional impact of cytotoxic pathological processes particularly strong.

## 5. Molecular Determinants of NMJ Structure

The structural features of human (and other) NMJs clearly make them targets of a variety of pathological processes. Knowledge of the molecular basis of how those structural features develop and are maintained might help efforts to counteract those processes.

### 5.1. Presynaptic Features—NMJ Size and Shape

Numerous studies have been made of the changes in NMJ size and shape which occur in response to experimental treatments which resemble some disease states. Most notable of these changes results from the outgrowth of the motor axon followed by differentiation of the underlying postsynaptic membrane [35]. What molecular processes might initially evoke axonal sprouting and then limit it?

The only well-identified “sprout-inducing” factor is the activity of the muscle cell itself. A range of studies in mammals show that blocking activity leads to the formation of sprouts of the motor axon which grow away from the original NMJ and often establish new, “ectopic”, sites of synaptic contact. For example, preventing quantal release from the terminal with botulinum toxin [42,43,44], or blocking ACh action on the muscle with α-bungarotoxin [45], both induce sprouting from the nerve terminal. What might be the molecular pathways by which activity controls the spatial properties of NMJs?

Inactivity, such as that caused by denervation, is known to cause the enhanced expression all along the length of muscle fibers of a number of molecules which are normally specifically concentrated at the NMJ. These include AChRs [46], Na_V_1.4 [47,48] and the nerve cell adhesion molecule NCAM [49,50,51]. NCAM is a member of the Ig-G superfamily of adhesion molecules and is known to influence neuronal morphology in vitro. Overexpression of NCAM in mice results in de novo sprouting from about 20% of motor nerve terminals [52]. This is consistent with the notion that molecules on the muscle cell surface play an important role in regulating the pattern of nerve growth. The normal suppression of NCAM expression away from the NMJ by muscle activity may act to restrict later axon growth once effective transmission has been established during development.

Agrin, a proteoglycan secreted from motor nerve terminals which triggers differentiation of the postsynaptic region, is also concentrated at the NMJ where it adheres to the synaptic BL even after muscle cell destruction [53,54]. Motor axons regenerating in vivo after nerve and/or muscle damage adhere selectively to the synaptic BL, even in the absence of muscle fibers [55]. Since axons extending from motor neurons growing in vitro also adhere relatively selectively to non-neural cells expressing agrin, it is likely that in vivo, agrin which binds to the synaptic BL after release from the nerve plays the role of a persistent axonal “stop signal” which subsequently restrains further growth [56].

These examples provide some insight into molecular factors which may influence the growth of axons on muscle cells. However, we are still far from a comprehensive understanding of the molecular basis of innervation patterns that could provide a satisfactory explanation of why, in general, electrically inexcitable muscle fibers have distributed innervation and electrically excitable ones do not.

### 5.2. AZ Density and Spacing

The small size of human NMJs is functionally significant because it is associated with a similarly small QC. At a structural level, this appears to arise from a constant spatial density of AZs at vertebrate, and even invertebrate, NMJs. This raises the question of the molecular factors that determine AZ spacing.

#### Structural Determinants of Quantal Content

As described above, the number of quanta released by a single nAP during low frequency stimulation (QC) is roughly proportional to the area of the NMJ. This proportionality reflects two factors; the relatively constant quantal output/AZ and the similarity of AZ spacing at different NMJs. The physiological factors governing QC/AZ are very complex and are considered in detail elsewhere [57].

**Figure 8:**
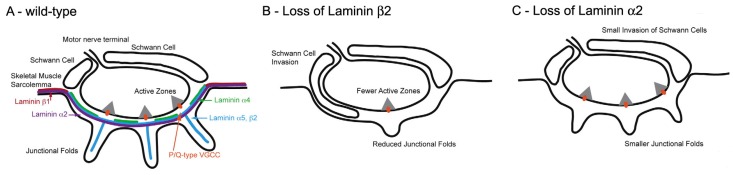
Laminins at the mammalian NMJ. Diagrams of the distribution of various laminin forms at normal and abnormal mouse NMJs. (**A**) Wild type; (**B**) Loss of laminin β2; (**C**) Loss of laminin α2. Note that in the absence of laminin β2, there are almost no folds and the Schwann cell extends into the synaptic cleft. The loss of laminin α2 causes similar, but less dramatic changes. (Adapted from Rogers and Nishimune, 2016, with permission) [67].

The molecular basis of AZ spacing remains poorly understood. One clue is the fact that in mutant mice lacking laminin β2, a BL component which is normally restricted to the synaptic cleft, the number of AZs is greatly reduced (Figure 8) [58]. This is associated with reduction of the QC to 50% of its normal value in mice [59] and a severe form of CMS in humans [33]. Biochemical approaches show that laminin β 2 binds to a complex of several molecules in the AZ, including the Ca_V_2 channels [60,61].

While these studies show the importance of laminins, and laminin β 2 in particular, for the presence and distribution of AZs, they do not lead to a detailed understanding of the quantitative features of AZ density and distribution. For example, the arrangement and density of different laminin molecules within the synaptic BL has not been determined.

### 5.3. Postsynaptic Fold Organization

Human NMJs are notable for the extensive folding of the postsynaptic membrane. While folding is a feature of most vertebrate NMJs, its prominence at humans NMJs may help to compensate for the small size and low QC. The apparent functional role of the folds arises from two spatial features; their depth and spacing, and their molecular organization.

#### 5.3.1. Fold Depth and Spacing

Folds arise normally during the maturation of the mammalian NMJ. In rats and mice, this occurs during the first few weeks after birth. In newborn mice, each NMJ is innervated by several motor axons, each of which can elicit effective neuromuscular transmission [62]. While this means that there is already a functional transmitter release mechanism and a prominent high-density cluster of AChRs, there is little folding of the postsynaptic membrane [63]. The maturation of the NMJ during the first postnatal month includes the increasingly focused accumulation of Na_V_1 channels within the depths of the growing folds [64]. Expression of the mRNA encoding Na_V_1s at the rat is first detectable at birth, and then increases greatly over the next month [65]. An attractive hypothesis is that the folds grow by the addition of new membrane which already contains Na_V_1 channels into the growing end of the folds, making them grow away from the region of high AChR density. It is also possible that adhesion between nerve and muscle cells to each side of the sites of transmitter release restricts the insertion of new membrane there, though again there is no direct evidence for this.

One class of molecules which are known to influence fold formation are the laminins (Figure 8) [11,66]. These heterotrimers consist of α β and γ chains and are components of the BL. One chain, laminin β2, is largely restricted to the BL at the NMJ where it plays an essential role in NMJ formation. Laminin β2-null mice are born live but never develop folds and have a much reduced number of AZs [67] (Figure 8B). Mice lacking another chain, laminin α2, also lack folds, but have normal, and normally located, AZs (Figure 8C). This suggests that the normal positioning of the AZs is not directed by the folds, and may arise autonomously within the nerve. In that case, it may be the positioning of the AZs that determines fold position. While the distinctive distribution of the various laminin chains at the NMJ may influence the development of the folds, and more generally of the NMJ, how laminin distribution is regulated remains to be established.

Whatever its molecular basis, there is clear evidence that the process of fold formation may be regulated very locally. In conditions where new synaptic contacts appear to be formed by axonal sprouts, regions of nerve–muscle contact with extensive folds (presumed original contacts) are commonly found adjacent to contacts with no folds (presumed new contacts) (Figure 7). This suggests that fold formation depends on very local interactions between individual nerve terminals and the underlying muscle fiber membrane.

#### 5.3.2. Ion Channel Distribution in the Folds

At the heart of the functional organization of the folds is the distinctive distribution of ion channels in the folded membranes (Figure 4). One aspect of that organization, the accumulation of AChRs at the crests of the folds, has received great attention over the years. This is largely because of the existence of a “near-perfect” ligand, α-bungarotoxin [3,54]. Elegant EM immunochemical studies showed that the depths of the folds contain a high concentration of the Na_V_1 channels that mediate the mAP, with a sharp boundary between them and the adjacent AChRs [19]. Underlying the folded postsynaptic membrane at the mature mammalian NMJ is a highly differentiated network of cytoskeletal proteins. Certain of these proteins are restricted to, or highly increased, in the crest of the folds (e.g., rapsyn, utrophin, syntrophin) [3,54] while others are concentrated in the depths of the folds in association with the Na_V_1 channels (e.g., β-spectrin, ankyrin G) [64,68]. While this arrangement of proteins may help to create and maintain the two distinct ion channel domains in the folds, it is not at all clear how the distribution of these accessory proteins is regulated.

One clue to the way in which the ion channel distribution arises is given by the apparently inherent tendency of AChRs to aggregate within the plane of the membrane to form clusters. A “plaque” of high AChR density is present at the immature mammalian NMJ some days before the increase in Na_V_1 expression begins. From the outset, the AChR plaque is associated with a network of cytoskeletal proteins (e.g., rapsyn, utrophin). It is possible that, as new membrane containing Na_V_1 channels is inserted into the postsynaptic membrane, the Na_V_1 molecules, and any associated cytoskeletal proteins, are excluded from the AChR plaque by some form of steric mechanism. In that case, the timing of expression of the different postsynaptic molecules might play a key role in determining their final location.

## 6. Concluding Remarks

The structural features of the NMJ set the spatial scene in which the molecules that mediate the events of transmission play out their roles. As such, they have an important impact on the efficacy of transmission. Human NMJs seem to occupy one end of a spectrum of vertebrate NMJ “plans”. Thus they are smaller, and have more extensive postsynaptic folds, than any other vertebrate species that has been studied. These features are important because they have clear functional significance. NMJ size is an important determinant of how much transmitter is released, and the spacing and depth of the folds appears to have an important effect on the amplification of transmitter action.

Both the spatial extent and intensity of folding at mature NMJs appears to arise by self-limiting processes. During development, growth is favored but as maturation progresses, the growth is limited as the mature form is achieved. We currently have almost no idea of the molecules and processes which regulate the formation and maintenance of these important structural features of the NMJ. It is clear that activity plays a role, presumably by triggering the limitation of growth once physiologically adequate activity has been achieved. However, the pathways from activity to growth-limitation are not well-understood. One activity-regulated molecule that may play an important role in axon growth is NCAM, but many others are likely to be involved. Almost nothing is known about the factors that regulate fold formation.

Identifying the molecular processes which determine the structural features of mature NMJs is technically very challenging. Although the formation of functioning NMJs in vitro is relatively simple, there are almost no systems available in which these contacts acquire anything like the structural properties of mature NMJs as they occur in vivo. It thus seems likely that analysis of naturally occurring and engineered mutations of NMJ molecules which have an impact on folding will be the most informative approach in the near future. A complementary approach would be to take advantage of genomic information that might shed light on the evolutionary trends in vertebrate NMJ evolution illustrated in Figure 6.

One “non-molecular” factor that seems likely to play an important role in determining molecular distribution within the NMJ is the timing of gene expression. The clearest example of this is seem in the maturation of the postsynaptic membrane, where expression of the genes encoding Na_V_1 channels follows that of AChRs by a considerable time. By the time Na_V_1 channels appear at the NMJ, the plaque of AChRs may be part of a highly structured molecular matrix which has the effect of excluding new ion channels with their associated cytoskeletal proteins. Similar effects of developmental timing may regulate other aspects of NMJ development.

While the factors that determine NMJ structure are of great inherent interest, it is their impact on NMJ function, and its impairment in disease, that make them clinically important. It is clear that many diseases in which neuromuscular transmission is impaired have associated abnormalities in NMJ structure. A better understanding of the factors which regulate that structure might open new avenues for treatment of patients suffering from these conditions. This provides an important incentive for future studies of the molecular basis of the structure of human NMJs.

## Abbreviations

NMJ	Neuromuscular junction
SVDOAJ	Synaptic vesicle
ACh	Acetylcholine
AChR	Acetylcholine receptor
AP	Action potential
nAP	Nerve action potential
mAP	Muscle action potential
EPP	Endplate potential
mEPP	Miniature endplate potential
QC	Quantal content
AZ	Active zone
Ca_V_	Voltage-gated calcium channel
Na_V_	Voltage-gated sodium channel
AChE	Acetylcholinesterase
NCAM	Nerve cell adhesion molecule
BL	Basal lamina
